# Recommendations for HLA Genotyping Data Standards and Clinical Laboratory Staffing Considerations

**DOI:** 10.1111/tan.70725

**Published:** 2026-04-20

**Authors:** Eric Spierings, Nicholas K. Brown, Katy Latham, James Robinson, Mark Melchers, Medhat Askar, Gerald P. Morris, Eric Weimer, Martin Maiers, Abeer Madbouly

**Affiliations:** ^1^ Matchis Foundation Leiden the Netherlands; ^2^ Laboratory for Translational Immunology and Central Diagnostics Laboratory, University Medical Center Utrecht the Netherlands; ^3^ Department of Pathology and Laboratory Medicine University of Pennsylvania Philadelphia Pennsylvania USA; ^4^ Histocompatibility and Immunogenetics, NHS Blood & Transplant London UK; ^5^ Anthony Nolan Research Institute London UK; ^6^ Royal Free Hospital, University College London Cancer Institute London UK; ^7^ World Marrow Donor Association‐WMDA Office Leiden the Netherlands; ^8^ Health Sector and College of Medicine, Qatar University Doha Qatar; ^9^ Department of Pathology University of California San Diego California USA; ^10^ Department of Pathology and Laboratory Medicine The University of North Carolina at Chapel Hill School of Medicine Chapel Hill North Carolina USA; ^11^ CIBMTR (Center for International Blood and Marrow Transplant Research), NMDP Minneapolis Minnesota USA; ^12^ MHC Therapeutics Bioconsulting Minneapolis Minnesota USA

## Abstract

The rapid advances in HLA genotyping technology and the massive amounts of associated data have created a demand for better and more efficient laboratory data management practices. However, while some standards have been developed, there is a need for comprehensive guidelines that include all laboratory data‐related processes such as messaging, storage and retention, documentation, reporting, validation and quality control. An important consideration in developing these recommendations is the feasibility of application in a laboratory setting without posing a substantial staff and cost burden for implementation and long‐term maintenance and the availability of publicly available tools. This article presents evidence‐based recommendations for multiple laboratory general data practices, focusing on HLA genotyping data and associated meta‐data. These recommendations are compiled by experts in the fields of histocompatibility and immunogenetics (H&I) and representation from multiple H&I worldwide professional society leadership with the long‐term goal of adopting these recommendations in future laboratory accreditation requirements.

## 1 Introduction

In the era of high‐throughput next‐generation sequencing (NGS), and to cope with multiple clinical requirements, genotyping technologies and ever‐changing regulatory standards, laboratories executing HLA genotyping must be able to manage a broad array of data types, each of which plays essential roles in the clinical interpretation. These data extend beyond the raw sequencing information that identifies the specific HLA alleles. Laboratories must be equipped with the necessary pipelines that incorporate genotyping instruments, reference sequences and analysis software packages to routinely and reliably produce HLA data used for clinical and/or research applications. Moreover, laboratories must be able to properly store and retrieve the data, and communicate this data in various ways and to multiple stakeholders such as legal and regulatory entities with data governance and compliance requirements, transplant centers, organ allocation organisations or stem‐cell registries, while maintaining operational efficiency and complying with various regulatory standards. This manuscript reports a set of evidence‐informed recommendations that focus on molecular HLA genotyping workflows in accredited laboratories, where HLA alleles are determined through DNA‐based typing, the different levels of complexities of the data and the proposed standards to address these complexities. Of note is that the proposed standards do not apply to imputation‐based or serology‐based methods, which lack underlying sequencing data and are commonly used in population or Genome‐wide association studies (GWAS). Such inferred HLA assignments do not meet the traceability and reproducibility requirements addressed in this work and typically used in a clinical setting.

Different HLA genotyping data aspects have their specific individual characteristics, levels of detail, and distinct requirements for how they are processed and stored. Whether it is the genotype data itself that must be accurately processed and communicated to inform clinicians to support their decision‐making, the metadata that supports interpretation, reproducibility and transparency of the genotyping process, or personally identifiable information (PII) that requires careful management to protect patient privacy, each type of data demands solid and potentially unique protocols for handling and storage. Additionally, there is regulatory data that ensures laboratories meet accreditation and legal requirements, as well as other ancillary types of information necessary for ongoing quality improvement and research.

For a laboratory to establish proper data‐centric practices, interact with internal and external stakeholders and develop the needed infrastructure without significant resource constraints, two main components are needed:

1. Universal standards for every stage in the data lifecycle, and
2. Publicly available tools to implement said standards.

The successful management of the diverse laboratory data types is critical for ensuring that clinical HLA laboratories can deliver reliable, reproducible and secure genotype results. Failure to properly handle any type of data can compromise the entire workflow, impacting the laboratory, the clinics and the patient. In the absence of established standards, multiple isolated processes are created to accommodate certain laboratory practices, sometimes created without consideration for proper documentation of how and why these processes were created necessary for reproducibility, compatibility with other institutions and long‐term consistent operations, maintenance and improvements or upgrades when new technologies are introduced. This can lead to long‐term added overheads and significant staffing and operational costs as well as loss of data and possible inconsistent clinical results, which impedes technical advances and optimisation and frustrates reproducible research. For instance, messaging standards such as the Histoimmunogenetics Markup Language (HML) [1] format and the Health Level Seven (HL7) Fast Healthcare Interoperability Resources (FHIR) [2] standard ensure timely, consistent and accurate communication between stakeholders, while storage and maintenance standards such as standardised file format, version control for traceability, and automated checks for data integrity guarantee data integrity over time. Challenges such as incompatible systems, lack of structured formats and insufficient documentation can impede clinical outcomes and collaborative research. Minimal standards serve as a universal baseline applicable across fields such as solid organ transplantation (SOT) and haematopoietic cell transplantation (HCT), spanning the entire spectrum from basic research to clinical application and bridging geographical regions. Enforcing standards without proper accessible tools and training resources can impede the application of these standards and pose a significant cost and logistical challenge to laboratories. The absence of universally available tools, such as HML or Minimum information for reporting next generation sequence genotyping (MIRING) [3] validation and conversion tools [4, 5] leaves gaps filled by inconsistent implementation of multiple ad hoc local interpretations with limited scope and applications, which, in the long run, can also add to the cost and overhead of laboratory operations. Our recommendations reflect evidence‐informed consensus recommendations among global experts in the field of histocompatibility and immunogenetics (H&I) with expertise and representation from the scientific community, professional societies, donor registries and regulatory organisations for SOT and HCT. They were developed based on evidence derived from published literature and experience obtained from clinical and research laboratory practices, and operating multiple modalities of laboratory data and software platforms. All presented recommendations are structured in three levels that reflect increasing degrees of data‐management maturity and associated resource requirements, rather than strict conceptual importance, and are listed below:

1. *Minimal Recommendations*—These are the minimal and necessary requirements needed to establish fundamental and robust laboratory data practices and are *strongly recommended*;
2. *General Recommendations*—These are comprehensive recommendations that are needed beyond the fundamental level of maintaining robust data practices and are generally *recommended* when there is the ability to include them;
3. *Optional Recommendations*—These are additional recommendations that are *encouraged* to be implemented for additional levels of data robustness and security.

## 2 Data Messaging and Communication Standards

**FIGURE 1:**
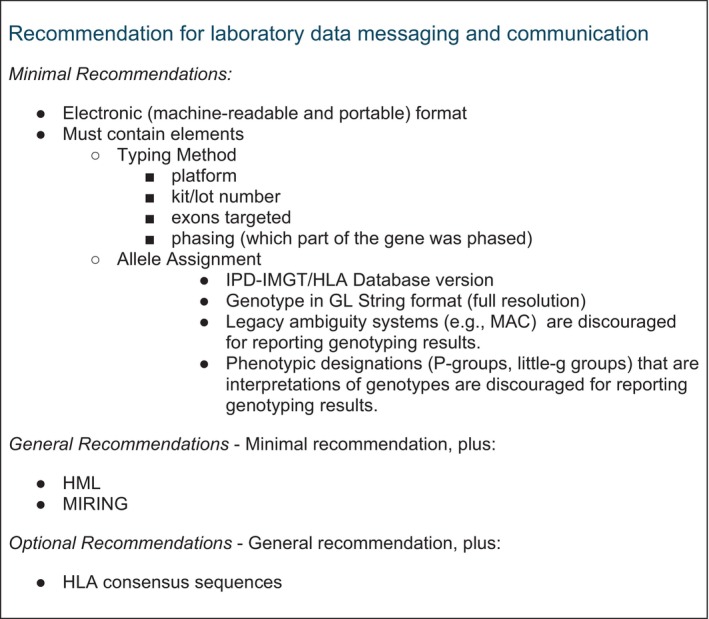
Recommendations for laboratory data messaging and communication. Minimal and general recommendations are not mutually exclusive; the general recommendations expand on and add context to the minimal requirements. Many minimal recommendations align with parts of the HML/MIRING standards. The same principles apply for the general recommendations in relation to the optional recommendations.

Several key data standards or recommendations have been established for handling HLA genotyping data, each with unique features and applications. Understanding these standards is vital for laboratories to adopt best practices and remain compliant with clinical and regulatory requirements. The following recommendations for laboratory data messaging and communication are summarised in Figure 1.

### 2.1 HLA Nomenclature

In terms of data standards, the field of HLA is fortunate to have a well‐established, published nomenclature [6]. This nomenclature allows communication of HLA designations for nucleotide or protein sequences between different parties. The HLA nomenclature assigns a unique allele name identifier, for example, HLA‐A*01:01:01:01 or a unique allele identifier (ID), that is, HLA00001. Whilst these identifiers can be used, they can become problematic depending on how the data is generated or intended to be used. In particular, HLA types for ambiguous sequence results have led to an additional tier of nomenclature‐style encodings to allow storage of long strings of alleles. These methods include the official HLA Nomenclature G and P groups, as well as the NMDP Multiple Allele Code (MAC) system [7], and the unofficial but often used little ‘g’ groups [8]. It should be noted, however, that these latter two unofficial nomenclatures are not consistently defined across laboratories or publications. Different groups have applied their own interpretations of ‘little g’ groups, which can lead to discrepancies. For this reason, the use of unofficial groupings is discouraged as they are interpretations rather than accurate reportings of HLA typing data. Both the European Federation for Immunogenetics (EFI) [9] and American Society for Histocompatibility and Immunogenetics (ASHI) [10] standards indicate that only the official World Health Organization (WHO) HLA Nomenclature should be used for reporting. Therefore, whenever HLA data are communicated, it is essential to rely on a standardised reference system so that results are reported consistently and understood uniformly across laboratories.

### 2.2 Reference Sequences in the IPD‐IMGT/HLA Database

Reference sequences are critical to the effective implementation of HLA data standards, forming the backbone for accurate interpretation, reproducibility and data exchange. In HLA genotyping, reference sequences provide a framework for comparing observed genetic variations to a standardised baseline, allowing the identification and reporting of alleles in a consistent and interpretable manner. The use of robust, well‐defined reference sequences is especially crucial as the field increasingly relies on advanced Next Generation Sequencing (NGS) technologies that generate high‐resolution, complex datasets requiring precise interpretation. Without reference sequences, the utility of HLA data standards would be significantly undermined, leading to inconsistencies and potential misinterpretations, particularly when describing ambiguities or novel polymorphisms in HLA genotyping data. A well‐known ambiguity is the pair HLA‐DRB1*14:01 and HLA‐DRB1*14:54, which are identical in their exon 2 DNA sequence. Typing at the antigen recognition domain level only (Exon 2 and 3 for class I alleles and exon 2 for class II alleles) would not resolve this ambiguity. Resolving this HLA‐DRB1 ambiguity will entail typing beyond exon 2 and comparison with the available reference sequences. Reference sequences allow these variations to be reported in relation to a well‐characterised baseline, adding clarity and context to the data. For example, when reporting novel alleles, reference sequences provide the necessary scaffolding to define their differences from existing alleles, making it easier for researchers and clinicians to understand their significance. This role is critical for data standards such as HML [1], which incorporate consensus sequences into genotyping reports, linking raw sequencing data to established reference sequences (see Section 2.4).

The primary reference resource for validated HLA reference sequences is the Immuno Polymorphism Database (IPD) ImMunoGeneTics (IMGT) HLA Database [11, 12]. The IPD‐IMGT/HLA database acts as the only official repository for sequences named by the WHO Nomenclature Committee for Factors of the HLA System [6]. The database provides validated and curated sequences representing each officially named allele. Data is provided in various formats and consumed by a wide range of users. Access to different releases is possible through differing branches on the GitHub repository. Each branch in the IPD‐IMGT/HLA GitHub repository corresponds to a specific IPD‐IMGT/HLA Database release (e.g., branch 3570 reflects version 3.57.0), allowing users and software systems to access the exact sequence and annotation data associated with that release. Further reference sets and commercial software often utilise these data. When doing so, they should clearly indicate the source (e.g., the IPD‐IMGT/HLA GitHub repository) and specify the relevant database release version. The use of standardised reference systems should exclude simplified or legacy systems such as NMDP MACs and the serologic ‘little‐g’ group designations, which lack sufficient resolution and reproducibility for interoperable data exchange.

The International HLA & Immunogenetics Workshops (IHIWs) have been instrumental in establishing guidelines for selecting and using reference sequences. During the 17th IHIW, participants adopted a centralised approach to managing HLA genotyping data, standardising the use of reference sequences [13, 14]. These reference sequences are used to contextualise genotypes, ensuring compatibility with data standards and facilitating interoperability across diverse platforms and laboratories [15].

The 18th IHIW further refined the guidelines for reference sequence selection, emphasising their role in supporting HLA data standards [16]. Community efforts, including the Data Standards Hackathon (DaSH) initiative [15, 17], have produced automated workflows for identifying and validating reference sequences for each IPD‐IMGT/HLA Database release [16]. These workflows ensure that selected references are comprehensive, accurate and reflective of the latest genetic knowledge.

### 2.3 GL‐String

Genotype List (GL) string is a gene‐agnostic, standardised format designed for representing and transmitting HLA genotyping results compactly and efficiently [18, 19]. The use of GL String facilitates communication between different laboratories, transplant centers and donor registries by providing a clear and concise way to convey HLA typing data at different levels of resolution. A significant advantage of GL String is its capability to encapsulate complex genotyping results, making them suitable for rapid data sharing, particularly in clinical settings where swift decision‐making is crucial.

The nomenclatures used to describe HLA alleles identify unique nucleotide and peptide sequences, along with expression patterns. However, these nomenclatures alone are often insufficient for describing the complete spectrum of genotyping results, especially when it comes to conveying ambiguities, relationships across loci and haplotype information. For example, the HLA nomenclature can identify the level of typing resolution by how many fields are being reported in an allele name (2‐field, 3‐field, etc.). However, no nomenclature rule can capture genotyping ambiguity when more than one allele call is reported in a genotype. The GL String grammar addresses this need by offering a structured method for documenting both known and unknown details about a given genotyping result.

Despite the utility of GL String, its accuracy is dependent on the version of the reference database under which they were created. To address this challenge, the GL String Code (GLSC) system was developed [19]. The GLSC system links each GL String with metadata that specifies the precise reference context—such as the gene‐family namespace, allele‐name code system and version of the reference database—in which it was produced and should be interpreted. This defined syntax allows GL String to be transmitted, parsed and interpreted in an unambiguous manner, ensuring that genotyping data is accurately understood within its specific context.

The incorporation of the GLSC system makes it possible to use GL String effectively on modern data systems, including those that employ HL7 FHIR [2]. This capability enhances the exchange of HLA and KIR genotyping data in a structured, consistent and contextually relevant format, supporting interoperability across healthcare platforms.

### 2.4 HML

HML [1] is an XML (eXtensible Markup Language)‐based standard [20] that has been specifically developed for the representation and transmission of HLA genotyping data. The advantage of HML over GL and GLSC is that its comprehensive and structured format also captures the critical contextual metadata necessary for accurate data interpretation. These metadata include essential details such as the sequencing platform used, the version of analysis software applied and the reference sequences utilised during the genotyping process. By encompassing this range of information, HML ensures that the data can be fully understood, valued and validated across different laboratory environments.

HML is flexible and allows laboratories to transmit HLA genotyping data in a machine‐readable format that is compatible with various software systems such as the HML gateway system at NMDP [21, 22] or other in‐house donor registry software. This capability supports seamless data sharing, reducing the risk of misinterpretation and eliminating the need for manual data input, which can be error‐prone and time‐consuming. Seamless data sharing implies data format consistency on the source and receiving end of the data, consistent data interpretation by both sides, and minimal to no data transmission errors. The use of HML promotes consistency in the way genotyping data is reported and communicated, enabling laboratories to integrate their data management practices more effectively with external partners, such as transplant centers and donor registries.

### 2.5 Minimum Information for Reporting Immunogenomic NGS Genotyping (MIRING)

The MIRING guidelines were developed to address the challenges of documenting and sharing the increasingly complex and detailed data generated by NGS technologies for HLA genotyping [3]. MIRING emerged as a collaborative effort led by the Immunogenomic Next Generation Sequencing Data Consortium (INGSDC), a group comprising histocompatibility clinicians, immunogenetic researchers, instrument manufacturers and software developers. This initiative was formalised during the 16th and 17th IHIW, with the guidelines tailored to ensure the consistent and reproducible reporting of genotyping data for clinical and research applications.

The driving force behind MIRING's development was the need for a robust standard to document every critical aspect of the genotyping process, including the instrumentation, software, reference sequences and contextual metadata. NGS technologies have revolutionised genotyping [23, 24, 25] by providing high‐resolution and phased data that overcome many of the ambiguities associated with older methods such as Sanger sequencing and sequence‐specific oligonucleotide probe typing (reviewed in [26]). However, the absence of standardised reporting formats hindered data interoperability and reproducibility, particularly when combining datasets across different laboratories or genotyping platforms. MIRING fills this gap by establishing a universal checklist for reporting NGS‐based genotyping results.

Unlike static reporting systems, MIRING is dynamic, allowing updates to genotype interpretations as reference databases and nomenclature evolve. This adaptability makes MIRING not only a tool for reporting current genotypes but also a future‐proof framework for re‐analysis as technology advances. HML versions 1.0 and later represent structured implementations of the MIRING guidelines, aligning the reporting format with the essential elements needed for transparent and reproducible HLA genotyping.

### 2.6 Data Communication

Within a laboratory, data communication involves interactions between various software systems, including sequence analysers, data analysis platforms and Laboratory Information Management Systems (LIMS). A critical step in this process is the ability to export HLA genotyping data from analysers and import it into laboratory systems efficiently and accurately. This ensures that raw sequencing data is effectively transformed into actionable results and documented in the LIMS or other reporting systems. Without such integration, laboratories may face bottlenecks in processing, increased error rates due to manual data entry and delays in delivering critical results.

Beyond the laboratory, the exchange of HLA data within the same institution and among institutions is equally important. Transplant centers and donor registries rely on timely, accurate, and interoperable data to identify suitable donor‐recipient matches and make informed clinical decisions [27, 28, 29, 30]. Similarly, inter‐laboratory data sharing is essential for quality control, collaborative research and validating novel findings. Standards such as HL7 [31] and HL7 FHIR [2] play a pivotal role in these interactions. FHIR, in particular, is designed for modern healthcare environments, enabling the secure transmission of complex HLA data—such as GL String, metadata and patient or center identifiers—alongside other clinical data within electronic health record systems [32, 33]. By facilitating real‐time data sharing, FHIR supports coordinated decision‐making across multidisciplinary teams and institutions.

### 2.7 The Importance of Data Identifiers for Data Manipulation and Communication

Accurate and complete HLA genotyping information is essential; however, the use of appropriate identifiers is equally critical. Identifiers that lack uniqueness or are prone to truncation without detection pose a significant risk of sample misidentification and related errors. It is not only necessary for an identifier to be unique within a laboratory or hospital, but in certain cases, it must also be globally unique. One such example is the ‘Global Registration Identifier for Donors’ (GRID), which serves as a globally unique donor identifier to facilitate accurate stem cell donor identification both in inter‐institutional communications and within individual institutions, as well as to ensure the correct identification of blood samples and stem cell products related to the stem cell donation and registration process [34]. The World Marrow Donor Association (WMDA) standards mandate that each registered stem cell donor be assigned a GRID [35].

Before the implementation of GRID as a mandatory identifier for donors throughout the entire transplantation process, multiple cases of donor misidentification occurred, including instances where donors were confused due to similar or truncated identifiers, leading to incorrect donors being used for transplantation or the danger of this happening. These errors resulted in severe clinical consequences, including transplantation mismatches that could have been prevented with the use of a globally unique identifier. A major incident that significantly contributed to the introduction of GRID involved the transplantation of a patient with cells from an incorrect donor who was a complete mismatch. This error resulted from the accidental truncation of the donor identifier, exacerbated by the variability in donor identifier lengths and formats, preventing easy recognition of the truncation. Consequently, the patient received a mismatched transplant, leading to severe clinical implications [36].

The implementation of GRID addresses these risks by ensuring that each identifier is exactly 19 characters long and includes a checksum, which significantly reduces the likelihood of undetected transcription errors [34]. Furthermore, the structured format of GRID makes it well‐suited for barcode scanning, which enhances accuracy by minimising human transcription errors and improving efficiency by enabling rapid, automated data capture and retrieval. This ensures that donor information is consistently recorded and accessed without discrepancies, reducing the likelihood of identification errors in clinical settings.

Having and correctly applying accurate and unique identifiers for HLA genotyping information is therefore an essential part of the data stored.

## 3 Data Storage and Retention

Establishing a robust process for data storage and retention in laboratories is crucial because it ensures the accuracy, reliability and integrity of clinical and scientific findings. Such processes prevent data loss, provide easy access to information for analysis, facilitate the reproducibility of experiments and ensure compliance with regulatory standards, especially since data integrity is critical for patient safety and research validity. Importantly, proper data management allows labs to validate their research and/or clinical results and make informed decisions based on reliable data.

**FIGURE 2:**
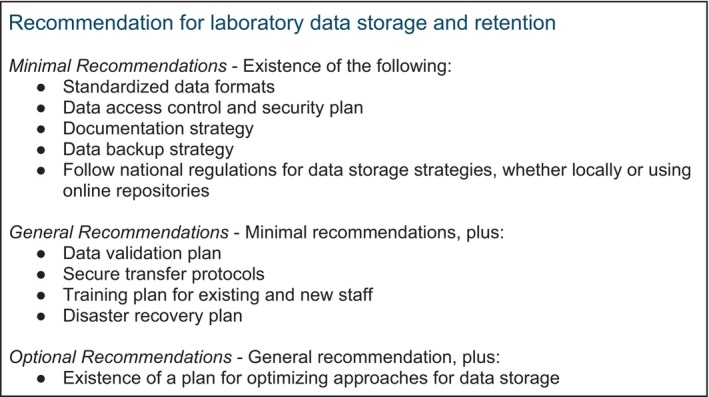
Recommendations for laboratory data storage and retention. Data backup is included as a Minimal Recommendation because loss of primary data is irrecoverable and a basic backup strategy is a prerequisite for any laboratory information system, whereas a formal, documented data validation plan is placed under General Recommendations because it typically requires additional informatics infrastructure and staff training.

The laboratory process for data storage and retention should include standardised file formats, clear data ownership and access controls, robust data validation procedures, secure transfer protocols, proper documentation of data transfers and compliance with relevant regulatory standards; ensuring data integrity, confidentiality and traceability throughout the transfer process, with specific attention to sensitive patient PII if applicable [37]. The recommendations for laboratory data storage and retention are summarised in Figure 2.

### 3.1 Data Formats and Data Standards

A wide range of data formats and exchange mechanisms are used across laboratories and information systems to handle HLA genotyping data. It is essential to distinguish between general‐purpose data structures and domain‐specific standardised formats. General data formats such as XML, JavaScript Object Notation (JSON) and Comma‐Separated Values (CSV) serve as syntactic containers but do not, by themselves, define how HLA data should be structured or interpreted. In contrast, standardised data formats such as HML (built on XML) and GLSC, which may be represented within JSON‐based data structures, provide structured, domain‐specific frameworks for encoding HLA genotyping results. Similarly, FASTA [38] and FASTQ [39] formats are widely used in sequencing workflows to represent nucleotide data, though they are not specific to HLA. The HL7 messaging standard provides an overarching healthcare data exchange framework, into which HLA‐specific content can be embedded using extensions such as FHIR. Table 1 summarises common data formats and standards, noting their primary use cases and their relevance to HLA data exchange. Several alternative methods for digitalising HLA genotyping data are discouraged, as they increase the risk of transcription errors, lack standardised structure and hinder automated processing (see Table 2).

**TABLE 1:** Summary of common data formats and standards, noting their primary use cases and their relevance to HLA data exchange.

Data format/standard	Description	Common laboratory application
XML	Extensible markup language	Basis for HML or structured HLA data exchange
CSV	Comma‐separated values	Storing tabulated data
JSON	JavaScript Object Notation	Used for WMDA Search, Match & Connect interactions and for importing data from analysers into LIMS
FASTQ	Text‐based format for storing raw sequencing data and quality scores	Used in NGS‐based sequencing workflows
FASTA	Text‐based format for reporting sequences only	Used for listing HLA sequences in the IPD/IMGT‐HLA Database
HL7	Standard for exchanging healthcare information	Used in HL7 FHIR Genomics Reporting Implementation Guide for histocompatibility and immunogenetics

**TABLE 2:** Examples of discouraged exchange media within the HLA field with reasons why to avoid them.

Document format	Issues
Spreadsheet formats	If not handled properly, spreadsheet programs automatically alter the formatting (e.g., date conversion) or corrupt data, particularly HLA data
PDF (portable document format)	Not easily machine‐readable; difficult to extract structured data
VCF (variant call format)	No support for raw sequencing reads; dependent on reference genome
Screenshots/scans	Data is image‐based and not machine‐readable without Optical Character Recognition (OCR) software
Hand‐written files and documents	Not machine‐readable; prone to interpretation errors and manual entry mistakes

### 3.2 Access Control and Data Ownership

To enhance data security and ensure regulatory compliance, laboratories should implement role‐based access control (RBAC) tailored to HLA typing workflows.

Specific roles can include:

*Data Owner* (Laboratory Director): Accountable for the entire data lifecycle, establishing access policies, approving data sharing agreements and ensuring Clinical Laboratory Improvement Amendments (CLIA), College of American Pathologists (CAP) and Institutional Review Board (IRB) compliance.

*Data Steward* (Technical Supervisor/Quality Manager): Ensures data quality through result validation, ambiguity resolution and maintenance of Standard Operational Procedures (SOPs). Reviews data before submission to registries or transplant centers.

*Data Custodian* (Bioinformatics Specialist/Information Technology (IT) Administrator): Manages technical infrastructure, database administration, backup systems, security controls and audit logs.

Implement Tiered Access Levels can include:

- *Administrator*: Full access to modify configurations, permissions and archived data (Laboratory Director, designated IT personnel). Includes ability to override locks on validated results with full audit trail.
- *Specialist/Expert*: Can review, interpret, validate results, resolve ambiguities, approve reports and submit to registries (certified laboratory scientists). Cannot delete validated results or modify permissions.
- *End‐User*: Read‐only access to finalised reports (transplant physicians, coordinators, search staff). Cannot access raw data or intermediate files

Additionally, laboratories should conduct annual access control audits to ensure compliance with accreditation standards (CAP, ASHI, EFI) and adapt permissions as staff roles evolve.

### 3.3 Data Validation Procedures

To ensure data accuracy and reliability, establish and perform data validation checks, such as range checks, logical consistency checks and outlier detection, to identify and address errors before transferring data. Examples of these validation measures include compliance of HLA data with WHO HLA nomenclature standards and reference sequences as well as proper compliance with the reporting genotype and file formats such as GL‐String and HML formats.

Additionally, documenting data validation procedures and their outcomes is necessary to maintain transparency and support auditing and accreditation processes by providing a clear record of how data is validated, the rules used and the results, allowing for verification and accountability. Example elements to include in the documentation of validation procedures include the purpose of validation, data sources, validation rules, tools and methods used and the frequency of performing validation processes. Importantly, documentation of validation outcomes is key for laboratory process consistency and improvement. Elements to document include pass/fail validation results, error types, corrective actions and exceptions that require special handling.

### 3.4 Secure Transfer Protocols

To safeguard sensitive information during transmission, it is important to utilise encrypted data transfer methods and implement secure network protocols and firewalls to prevent unauthorised access and ensure data security. Additionally, maintaining detailed records of all data transfers, documenting the date, time, transferring user, recipient, data type, and any associated validation results is needed to track the data, particularly for problem handling. Including clear transfer instructions and procedures within the documentation is key to ensuring accuracy, transparency and accountability.

### 3.5 Compliance With Regulations

European laboratories abide by General Data Protection Regulation (GDPR) [40] and the International Organization for Standardization (ISO) 15189:2022 [41] standard, which refers to the following general principles:

- Data should be anonymised when feasible, for example by using a unique laboratory identifier during test processing, which is separately linked to the patient in the central LIMS.
- Access to the LIMS should be role‐based, ensuring that users only have access to functions and data necessary for their responsibilities within the laboratory.
- Retention periods for records should be defined, taking into account the purpose of the test, applicable regulatory requirements and national laws.
- All records, regardless of format, must be preserved and retrievable in line with the defined retention and access policies.

The United Kingdom [42] and European Union regulations [40] are aligned in factors to protect PII, including consent to test and store for intended purposes, limiting access to information, only storing information relevant, regular staff training and audit and regular review of policies. The WMDA defines standards that apply to member organisations that want to be certified [35]. The standards defined in Chapter 5 concern information technology and information management. Compliance with Chapter 5 in the WMDA standards is therefore essential for the protection of PII of both patients and donors.

In the United States, data privacy regulations like HIPAA and state‐level laws, such as the California Consumer Privacy Act (CCPA) [43], provide a framework for protecting individuals' personal information. Health Insurance Portability and Accountability Act (HIPAA) Privacy Rule, for example, regulates how health information is used and disclosed by different entities and requires covered entities to retain certain compliance‐related documentation, like policies and procedures, for a minimum of 6 years from the date of creation or last in effect, whichever is later [44]. State laws may also mandate longer retention periods, and it's crucial to comply with the more stringent requirement.

Adhering to these regulations helps maintain data security, integrity and legal compliance. Protecting patient privacy by implementing de‐identification protocols to remove or mask all PII before data transfer ensures compliance with privacy regulations and reduces the risk of unauthorised access.

### 3.6 Data Backup and Recovery

Establishing a reliable data backup process prevents data loss due to system failures or unforeseen events. A single or mix of backup methods can be in effect depending on the sensitivity of the data and urgency of recovery, including local or network storage devices, cloud storage or online backup services. Laboratories should design a protocol for the frequency and strategies of data backup for different levels of data sensitivity/importance (such as patient data, LIMS data, system configuration files, etc.). Backup strategies can include full, incremental or differential backup approaches.

### 3.7 Training and Awareness

Providing ongoing training to laboratory staff on data handling, security protocols and compliance requirements ensures proper data management and fosters a culture of responsibility and vigilance to promote adherence to best practices and regulatory standards. It is advisable for laboratories to develop policies and continuity plans for staff onboarding and continuous training to update their staff on state‐of‐the‐art laboratory data standards and recommendations for effective laboratory practices, their importance and impact of excluding these standards.

Improper record retention in laboratories can lead to a range of consequences including compromised patient safety, legal issues, difficulty in quality control, inability to reproduce results, compliance failures, loss of accreditation and/or contractual agreements, reputational damage and potential for inaccurate diagnoses due to missing or unreliable data, making it crucial to maintain accurate and complete laboratory records as per regulatory standards [45].

Without proper documentation, critical information about patient samples or test procedures might be missing, leading to potential misinterpretations or incorrect treatment decisions. Plus, failing to adhere to regulatory requirements regarding record retention can result in fines, sanctions and loss of laboratory accreditation.

Incomplete or missing records can disrupt workflow, increase operational costs and make it difficult to monitor and maintain quality standards within the laboratory, potentially impacting the accuracy of test results. Consequently, absence of complete records or proper documentation can make it difficult for clinicians to track discrepancies in test results and researchers may struggle to replicate experiments or findings, hindering scientific progress and in extreme cases of a medical malpractice lawsuit, incomplete or inaccurate records can significantly weaken a laboratory's defence and may lead to public scrutiny and damage to laboratory credibility.

## 4 Practical Implications

### 4.1 Urgent Need for Using HLA Data Standards

The urgent need for using HLA data standards stems from the increasing reliance on HLA genotyping in critical medical fields such as transplantation, immunotherapy and population genetics. Without the adoption of robust and universally accepted standards, the short‐ and long‐term consequences can be significant. In the short term, the absence of data standards leads to inconsistent reporting and interpretation of genotyping results, which can delay clinical decisions, particularly in time‐sensitive situations like organ transplantation. Miscommunication between laboratories and transplant centers can result in mismatched donors and recipients, jeopardising patient outcomes. In the long term, the lack of standardised practices can create fragmented datasets, hindering collaborative research and the development of advancements in immunogenetics. It also risks the loss of valuable historical data due to incompatible formats or inadequate documentation. Furthermore, laboratories that fail to implement standards may struggle to comply with regulatory requirements, compromising their accreditation and operational sustainability.

### 4.2 Impact of Standards on Clinical Practice

The current HLA standards, while comprehensive, present both advantages and challenges for implementation in clinical practice, particularly in settings with limited resources or urgent operational demands. On the positive side, standards like HML and GL String offer robust frameworks for representing and exchanging HLA data, ensuring consistency, accuracy and interoperability across laboratories and clinical centers. They enable seamless communication of complex genotyping results, fostering collaboration and improving patient outcomes in transplantation and other HLA‐driven therapies. However, their full implementation can be resource‐intensive, requiring significant investment in software, training and data infrastructure, which may be impractical for smaller or less‐equipped laboratories. A practical, minimal approach—incorporating essential elements such as patient identifiers, center identifiers, HLA database version and a GL String—strikes a balance between functionality and feasibility. This minimal HML format ensures that the critical data required for patient care and clinical decision‐making are accurately transmitted while reducing the burden on laboratories. Such an approach maintains the benefits of standardisation while making implementation achievable for a broader range of clinical settings, ultimately promoting more equitable access to high‐quality HLA data management.

The recommendations here represent a streamlined adaptation of the full MIRING guidelines, designed to simplify reporting requirements while maintaining the essential elements needed for reproducibility and accuracy. This condensed framework focuses on the core components of MIRING, which are a machine‐readable description of the typing method and the allele assignment. This recommendation is a compromise so that laboratories with limited resources can still adhere to standardised reporting practices without the burden of excessive documentation. By retaining only the most critical information, this recommendation ensures that genotyping data remains interpretable and transferable across institutions while reducing the complexity and cost of implementation.

### 4.3 Current Clinical Practice

Clinical adoption of structured HLA genotyping exchange standards remains heterogeneous. Outside a few large networks and mandated pathways, many laboratories and transplant programs still rely on unstructured reports (often PDFs) and manual data entry, limiting interoperability and increasing transcription and interpretation risk. The three examples below are therefore not an exhaustive survey, but illustrate anchor points across key exchange pathways: (i) registry‐driven verification typing reporting at scale (NMDP), (ii) national registry–transplant center exchange (Matchis) and (iii) organ allocation network integration for deceased donor HLA data (Eurotransplant). Together, they show that standards‐based exchange is feasible and already operational where governance, incentives and validation infrastructure exist, while broader routine adoption across transplant centers remains incomplete. These practices matter because they enable automated validation, consistent representation of ambiguity and metadata, safer reuse of external typings in local LIMS environments and more reliable clinical decision‐making and downstream data reuse.

NMDP implemented HML as a standard for all contracted laboratories starting in 2007, including reporting of all ambiguities using GL String or NMDP multiple‐allele code and with documentation of the typing method and reference context, including a method description, platform, exons targeted, IPD‐IMGT/HLA Database version and reporting of raw data in the form of consensus sequences. This standard is based on predecessors that have been used for electronic reporting dating back to 1994. The adoption of HML as a standard for electronic reporting from clinical transplant center laboratories has had a much slower adoption, but as of late 2024, over 97% of all NMDP donor confirmatory typing results in the US are being reported electronically using the HML format.

**FIGURE 3:**
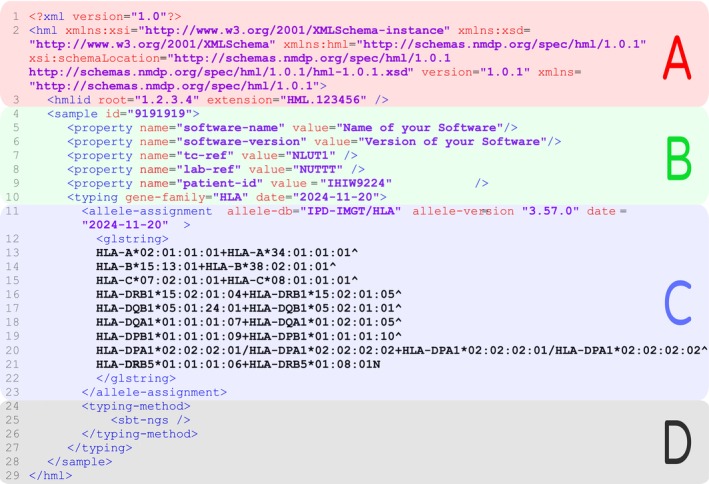
An example of a ‘stripped‐down minimal HML’ message structure illustrating the hml structure. Typing data relate to reference cell line IHW9224 and typing data were extracted from the IPD‐IMGT/HLA database, hence the lack of a typing method [11, 46]. The example includes essential elements for communicating an HLA typing. A typical HML follows an XML schema, with a header (block A in red) and a sample and/or patient identification section (block B in green). For correct communication of the origin of the typing and the registry‐specific identifiers, sample information was extended with properties for typing center <property name="lab‐ref" value="NUTTT" />, transplant center <property name="tc‐ref" value="NLUT1" /> and patient identifier <property name="patient‐ref" value="IHW9224" />. Subsequently, the HML reports information on the HLA typing (block C in blue), here represented as a GL String <glstring> preceded by the IPD‐IMGT/HLA Database version information <allele‐assignment allele‐db="IPD‐IMGT/HLA" allele‐version="3.57.0" date="2024‐11‐20">. Details on how to report the typing method (block D) can be found in the Supporting Information. These supplementary file also contain annotations regarding the Minimal, General and Optional Recommendations.

In November 2024, Matchis, the Dutch stem‐cell registry, launched a pilot program to digitise and streamline the reporting of confirmatory HLA typing data for stem‐cell donors and recipients in the Netherlands, using a minimal HML format (Figure 3). This initiative aims to replace the reliance on PDF files and typing essential data from these reports manually—a process that was prone to clerical errors and time delays—with a structured and standardised digital data format. A key feature of this system is its backend validation process, which includes automated checks for multi‐allele codes maintained by the NMDP donor registry [7]. This implementation bridges the discrepancy between the way GL String reports ambiguities and how the WMDA and registries deal with ambiguous typing data. The pilot program has demonstrated significant improvements in efficiency and accuracy and is set to be implemented nationally in 2025. In the first stage, Matchis aims to receive data from transplant centers. A second phase will focus on communicating registry typings of candidate donors to the transplant centers in HML format, allowing the transplant centers to include these external typings in their lab system for verification purposes.

The application of data standards to solid organ transplant is potentially more fraught, in that the application of the data relies on the interpretation of features (i.e., serologic reactivity) beyond the specificity of the HLA allele. While this has been practically applied by requiring laboratories to report HLA results in terms of these features (i.e., serologic antigens), this has become increasingly problematic with the application of HLA genotyping and particularly with the implementation of high‐resolution HLA genotyping. With this, the challenge becomes that gaps in the definitive assignment of features to genotypes (i.e., lack of defined serologic reactivity for more recently reported genotypes) or discrepancies in the interpretation (i.e., variations in reporting WHO‐defined serologic antigens versus IPD‐IMGT/HLA Database HLA Dictionary Expert‐assigned predictions of reactivity) can lead to ambiguities in reporting that are not evident depending on how data is shared. For example, reporting at the serologic split‐antigen level (the current requirement by ASHI [10], and CAP [47]) does not provide information on the specific allele present, which may have a significant impact on serologic reactivity. Similarly, the assignment of several split‐antigen level typings requires reporting data at the 2‐field level, which may be provided by low‐resolution typing methods sufficiently for the assignment of serologic antigen typing, but falsely implies allele‐level typing potentially leading to erroneous decision‐making. Currently, this gap is incompletely addressed in the United States by Organ Procurement and Transplantation Network (OPTN) requirements to share the HLA typing reports as a PDF file in addition to data entry for serologic typing [48]. This situation presents a significant risk for misinterpretation of data with potential clinical impact.

In January 2023, Eurotransplant, the transnational organ allocation organisation covering Austria, Belgium, Croatia, Germany, Hungary, Luxembourg, the Netherlands and Slovenia, implemented a minimal version of HML specifically for the digital exchange of deceased solid organ donor typings [49]. More than 30 HLA donor typing laboratories currently use the HML data standard in their communication with Eurotranplant [50]. This adaptation underscores HML's flexibility and scalability, showcasing how it could be tailored to fit specific organisational requirements while maintaining its core function of reliable and standardised data representation in a clinical setting.

### 4.4 Case Study: An Example for an HML Approach

**FIGURE 4:**
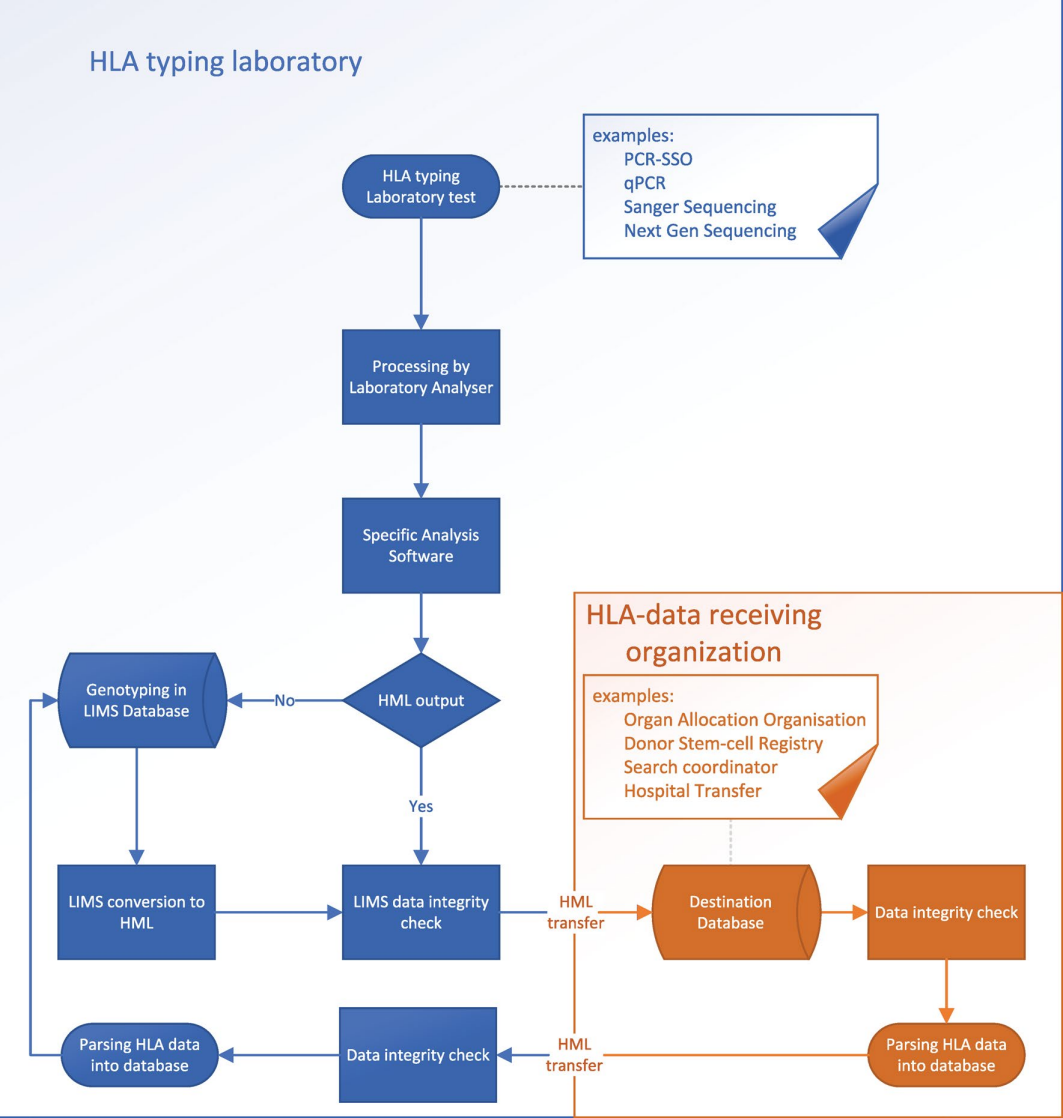
Data flow of HLA data in typing laboratories for implementation of HML.

Integration with LIMS or donor/recipient databases is a critical step in this process, as it forms the backbone of efficient data management in clinical laboratories and transplant registries. Collaboration with LIMS vendors and database constructors is essential to ensure seamless integration of HML‐formatted data, requiring the development of standardised pipelines for data import, storage and export. This process involves modifying existing LIMS and database architectures to support HML specifications, ensuring accurate parsing and structured storage of HLA genotyping data (Figure 4). Publicly available tools, such as the HML, miring‐validator and hml‐fhir‐app repositories maintained by NMDP [51], offer reference implementations and validators that support integration of HML and GL String data into clinical systems. These tools facilitate standard‐compliant adoption and can accelerate the development of interoperable interfaces.

A key challenge in this integration is addressing inconsistencies in data representation across different laboratory environments. Standardisation of HML formatting within database systems should involve automated validation processes that detect and correct errors in allele and genotype encoding. This validation process includes implementing real‐time error detection mechanisms to flag anomalies in data entry, thereby reducing manual corrections and improving overall efficiency. Additionally, robust data conversion modules should be incorporated to facilitate compatibility between legacy formats and the HML standard, ensuring a smooth transition for laboratories adopting this system. Automated quality control measures, including cross‐checking HLA genotyping results against reference databases such as the IPD‐IMGT/HLA Database, will help laboratories mitigate discrepancies and improve consistency in reporting.

Currently, HML is defined exclusively using the XML serialisation format. This stands in contrast to standards such as HL7 FHIR, which support both XML and JSON representations. As JSON continues to gain popularity due to its simplicity and widespread use in modern web and API development, it is increasingly important for HML to support a JSON‐based definition. Expanding HML to include a JSON representation would facilitate broader adoption across diverse platforms and systems. Notably, the underlying structure and semantics of HML need not change between XML and JSON formats, as these are simply alternative serialisation methods for the same data model. Scripts to convert XML to JSON and vice versa can be constructed to deliver reliable standardised tools, ensuring consistency and interoperability.

### 4.5 Considerations for Staffing to Adopt and Maintain Laboratory Data Standards

The successful adoption and maintenance of laboratory data standards necessitates strategic workforce planning, particularly in the inclusion of IT and bioinformatics personnel in laboratory staffing structures. These professionals play a critical role in managing data pipelines, ensuring compliance with data standards, integrating LIMS and maintaining secure and efficient data exchange protocols. Without dedicated IT and bioinformatics staff, laboratories risk inefficiencies, data mismanagement and difficulties in implementing new technologies.

A common challenge faced by laboratories is the lack of prior planning and budgeting for IT and bioinformatics staff. Many institutions primarily focus on laboratory personnel involved in wet‐lab procedures, underestimating the technical demands of data handling and standardisation. The absence of IT specialists can lead to costly ad hoc solutions, reliance on external vendors for technical support and increased risk of compliance failures. Moreover, the lack of an in‐house team capable of managing software updates, troubleshooting data inconsistencies and ensuring system interoperability can significantly hinder laboratory efficiency and responsiveness.

Failure to allocate resources for IT and bioinformatics staff has long‐term time and cost consequences. Laboratories without proper IT infrastructure often struggle with data integration challenges, leading to delays in reporting results, inconsistencies in data interpretation and increased workload for laboratory personnel. Retrospectively fitting IT support into an existing workflow is far more costly than incorporating it from the start. Additionally, without adequate staffing, laboratories may be unable to fully leverage automation, machine learning applications or real‐time data validation tools, further compounding inefficiencies.

Beyond daily operations, the absence of robust IT and bioinformatics support limits a laboratory's ability to engage in research and development. A well‐structured IT framework is essential for handling large‐scale datasets, participating in multi‐center collaborations, and contributing to advancements in HLA genotyping and immunogenetics. Without the necessary infrastructure and personnel, laboratories face significant barriers to innovation, limiting their role in shaping the future of the field. Investing in IT and bioinformatics staffing is, therefore, not only crucial for meeting current data standardisation needs but also for ensuring laboratories remain at the forefront of scientific and clinical advancements.

### 4.6 Path to Implementing Data Standards, Community Efforts and Stakeholder Roles

Our recommendations emphasise that robust data standards are essential to enhance efficiency, accuracy and interoperability in immunogenetics and histocompatibility practices. It underscores that the successful implementation of such standards requires the coordinated engagement of multiple stakeholders and governing bodies—including researchers, laboratory directors, professional societies, accrediting bodies and technology vendors. This multi‐stakeholder approach is supported by published literature, which indicates that effective standardisation can lead to significant improvements in data quality and clinical outcomes.

Central to the proposed recommendations is the role of the scientific community, whose responsibilities include conducting research, setting testing protocols and building the evidence base necessary to drive change. Immunogenetics scientists and histocompatibility laboratory directors are positioned to generate insights that inform and validate data standard practices. These efforts align with findings from recent studies that highlight the impact of evidence‐based guidelines on enhancing laboratory performance and patient care.

Professional societies and accrediting bodies—such as ASHI, the European Federation of Immunogenetics (EFI) and CAP—are charged with establishing guidelines, accrediting laboratories and ensuring compliance with best practices [9, 10, 47]. Their influence is pivotal in motivating healthcare institutions to adopt data standards, a process that is mirrored in broader healthcare systems where regulatory requirements have successfully driven technology adoption and standardisation. Importantly, accreditation organisations must take responsibility for ensuring that laboratories have access to affordable, standardised solutions, facilitating broad adoption without imposing excessive financial or logistical burdens. To ensure a smooth transition towards full compliance, accreditation organisations should initially introduce these data standards as advisory recommendations (‘should’) before gradually making them mandatory (‘must’) over time. This phased approach would allow laboratories of all sizes to adapt their infrastructure, allocate resources efficiently and receive necessary training, ensuring that compliance is feasible across diverse operational environments. By implementing a gradual transition, the EFI and ASHI can foster widespread adoption while minimising disruptions to clinical and research workflows.

The WMDA Standards establish the minimum guidelines required to support haematopoietic stem cell transplantation and cell therapies [35]. Compliance with these standards is strongly encouraged for stem cell registries. To further strengthen data integrity and interoperability, the WMDA Standards should be updated to mandate the storage and exchange of HLA genotyping data in standardised formats. By implementing these updates, the transplantation community can ensure that HLA genotyping data remain accurate, interpretable and reproducible across different laboratory environments and over extended periods.

To facilitate the adoption of these standards, stakeholders can propose changes to the WMDA Standards Committee. A formal proposal should outline the need for uniform data storage, ensuring that all HLA genotyping data are formatted consistently to enable long‐term re‐interpretation and validation. Establishing this requirement would drive demand for standardised data management solutions in HLA laboratories and create a sustainable ecosystem where genotyping results can be efficiently utilised for clinical decision‐making and research.

In addition, the integration of data standards into laboratory management systems and HLA typing software is discussed, noting the challenges arising from vendor competition and potential conflicts of interest. The interplay between vendors—ranging from laboratory information system providers to HLA typing software vendors—illustrates a complex, multidirectional network of interactions. Collaboration across vendor lines is crucial for fostering an environment conducive to standard adoption, despite competitive pressures. The proactive engagement of these vendors in the introduction of HML within the Eurotransplant community and their participation in the 17th, 18th and 19th IHIW underscore their commitment to supporting standardisation efforts. While third‐party reliance enables laboratories to adopt efficient, user‐friendly data management systems, it also presents challenges such as cost barriers.

Following the process of Eurotransplant, the OPTN needs to develop and implement tools for automated data transfer and integration into transplant‐related workflows. Establishing standardised formats such as HML and GL String, as those usable by OPTN‐managed resources, would support policies requiring the use of standardised data formats, streamlining data exchange between transplant centers, donor registries, and laboratories, reducing inconsistencies in reporting, and improving patient outcomes. This regulatory endorsement would also serve as a framework for US laboratories to develop infrastructure that aligns with global best practices.

Taken together, these recommendations present a comprehensive framework in which data standards implementation is viewed as a cascading process. It begins with the scientific community's efforts to build a robust evidence base, which in turn informs the guidelines set by accrediting bodies. These guidelines then drive institutions to demand compliant technological solutions from vendors, ultimately creating a unified and efficient system that enhances patient care and fosters innovation.

## Author Contributions

This position paper is the result of a collaborative effort by all listed authors. Eric Spierings and Abeer Madbouly initiated and coordinated the discussions and led the development and writing of the manuscript. They organised and facilitated the core discussions and wrote and structured the paper. Nicholas K. Brown, Katy Latham, James Robinson, Mark Melchers, Medhat Askar, Gerald P. Morris, Eric Weimer and Martin Maiers contributed actively and substantially to the discussions, formulation of key issues, and the writing and refinement of the text. All authors critically reviewed and revised multiple manuscript drafts and provided intellectual content essential to the final version. All authors have read and approved the final manuscript and agree to be accountable for all aspects of the work.

## Funding

This work was supported by the National Institutes of Health, R01AI128775.

## Conflicts of Interest

A number of authors currently hold positions on a number of committees within ASHI [Eric Spierings, Nicholas K. Brown, Eric Weimer, Abeer Madbouly], EFI [Eric Spierings, Katy Latham, James Robinson], UNOS and CAP [Gerald P. Morris], the IHIW [Eric Spierings, Martin Maiers] and WMDA [James Robinson, Eric Spierings, Martin Maiers, Mark Melchers, Abeer Madbouly], focused on data standards, bioinformatics, standards and regulations. Eric Spierings serves on the Scientific Committee (member) and the Bioinformatics & IT Committee (chair) of the European Federation for Immunogenetics (EFI), is liaison to the ASHI Science and Technologies Initiatives Committee (STIC) and holds editorial roles with HLA: Immune Response Genetics, Frontiers in Immunology and Cellular Immunology. He is co‐president of the International HLA & Immunogenetics Workshop Council. In the past 5 years, he has executed research projects funded by The Dutch Kidney Foundation, The European Union, the 18th International HLA & Immunogenetics Foundation, and PIRCHE AG. He received/receives in‐kind support for research projects from Werfen and CareDx. He serves as scientific advisor for PIRCHE AG and received speaker fees and/or travel compensation from Thermofisher, GenDx, PIRCHE AG and Miltenyi Biotec. All these activities were and are academic or collaborative in nature, and no personal financial benefit has been received. None of these roles influenced the content or recommendations presented in this manuscript. Nicholas K. Brown currently serves as a commissioner on the Accreditation Review Board, a member of the Board of Directors, the Annual Meeting Programming Planning Committee, and as a Laboratory Inspector for ASHI. He previously served as chair of the Quality Assurance and Standards Committee, as an associate editor for ASHI Quarterly, and on the Annual Meeting Abstract Committee. Dr. Nicholas K. Brown has acted as a scientific advisor for CareDx and has received speaker fees and/or travel support from Thermo Fisher Scientific, GenDx and Werfen. All activities have been academic or collaborative in nature and did not influence the content or recommendations presented in this manuscript. Eric Weimer is a member of the Board of Directors of ASHI. This role has not influenced the content or recommendations presented in this manuscript. Katy Latham is an examiner for the Royal College of Pathologists (RCPath) and represents the RCPath for professional appointments. She is the Chair of the EFI Standards and Quality Assurance Committee, is liaison to the ASHI Standards Committee, and is involved in a Global Standards working group representing EFI. For these roles, no personal financial benefit is received. She is a Technical Assessor employed by the United Kingdom Accreditation Service (UKAS) for ISO15189 and represents Histocompatibility and Immunogenetics on the UKAS ISO15189 Technical Advisory Committee, for which she receives an honorarium. None of these roles influenced the content or recommendations presented in this manuscript. James Robinson is employed by Anthony Nolan and holds honorary positions with University College London (UCL), and is a visiting scientist at EMBL‐EBI. He serves on the Bioinformatics & IT Committee EFI and is chair of the Bioinformatics and Innovation Committee of the WMDA. He is a member of the WHO Nomenclature Committee for Factors of the HLA System, the ISAG/IUIS‐VIC Comparative MHC Nomenclature Committee and works with a number of other related nomenclature committees in the immunogenetics and comparative MHC fields. He is a section editor for HLA: Immune Response Genetics and an editorial board member for Human Immunology. None of these roles influenced the content or recommendations presented in this manuscript. Mark Melchers is employed by the WMDA and contributed to this work while representing the interests of its community. This constitutes a potential conflicts of interest. He believes that his participation did not introduce bias. No other conflicts are declared. Gerald P. Morris is the current Chair of the OPTN Histocompatibility Committee and a Member of the CAP Histocompatiblity and Identity Testing Committee. His scientific roles include Section Editor for The Journal of Immunology and Member of NIH study sections. In the past 5 years, Dr. Gerald P. Morris has received research support from the NIH, ThermoFisher, CareDx, PIRCHE AG, and Werfen. Dr. Gerald P. Morris has received travel support from ThermoFisher and ASHI. These activities were all academic and no personal financial benefit was received. Dr. Gerald P. Morris is also a scientific advisor and holds equity in Immunomatics Inc. None of these roles influenced the content or recommendations presented in this manuscript. Martin Maiers is an employee of NMDP and a Senior Scientific Director for the Center for International Blood and Marrow Transplant Research. He serves on the editorial boards of Human Immunology, HLA: Immune Response Genes, Frontiers in Immunology, and Frontiers in Genetics. He is a member of the IHIW Council, a member of the WHO Nomenclature Committee for Factors of the HLA System, and a co‐founder and board member of the Society for Immune Polymorphism. Abeer Madbouly serves as the Co‐Chair of the Abstract Committee of ASHI and the Cell & Gene Therapy Committee of the WMDA and holds editorial roles with Human Immunology and Frontiers in Genetics. She is the founder of MHC Therapeutics Bioconsulting. None of these roles influenced the content or recommendations presented in this manuscript.

## Supporting information


**Data S1:** tan70725‐sup‐0001‐DataS1.zip.

## Data Availability

Data sharing not applicable to this article as no datasets were generated or analysed during the current study.
