# Supplementary material for: Recommendations for HLA Genotyping Data Standards and Clinical Laboratory Staffing Considerations
Source: HLA. 2026 Apr 20;107:e70725. doi: 10.1111/tan.70725 (PMC13094400; doi:10.1111/tan.70725)
Supplement: Supplementary file 1 — Data S1: tan70725‐sup‐0001‐DataS1.zip. [file TAN-107-e70725-s001.zip › tan70725-sup-0001-DataS1/tan70725-sup-0001-FigureS1.pdf]

```xml
<?xml version="1.0"
      encoding="utf-8"?>

<!-- This HML file provides a detailed example meeting the proposed recommendations, indicated as: -->
<!-- * MINIMAL RECOMMENDATION -->
<!-- * GENERAL RECOMMENDATION -->
<!-- * OPTIONAL RECOMMENDATION -->
<!-- See table/schema 1 in the main document for reference to the RECOMMENDATIONS -->

<!-- The details below have been limited to a single locus (HLA-B) for clarity -->
<!-- Annotations in this file are here for the sole purpose of explaining the content -->


<!-- MINIMAL RECOMMENDATION: Electronic (machine-readable and portable) format -->
<!-- This file follows the HML schema, which is an xml format. Details are listed in the first section -->
<!-- XML declaration specifying the XML version and character encoding used in this file. PLEASE NOTE: The line above MUST be the very first line in the HML and may not be preceded by comments. -->


<!-- GENERAL RECOMMENDATION: HML. Report here the used schema and the specific version -->
<!-- details on the xml schema -->
<hml xmlns="http://schemas.nmdp.org/spec/hml/1.0"
     xmlns:xsi="http://www.w3.org/2001/XMLSchema-instance"
     xsi:schemaLocation="http://schemas.nmdp.org/spec/hml/1.0 https://schemas.nmdp.org/spec/hml/1.0.2/hml-1.0.2.xsd"
     version="1.0.2"
     project-name="20260316_HLA">

  <!-- the hmlid gives this document a locally unique identifier -->
  <hmlid root="2.16.840.1.113883.19.3.999.5">
  </hmlid>

  <!-- the section below identifies the reporting center -->
  <reporting-center reporting-center-id="NUT">
  </reporting-center>

  <!-- the following section describes all aspects of the sample -->
  <!-- sample ID and the center-code of the center that executed the typing -->
  <sample id="123456789012" center-code="NUTT">
    <!--How was the sample collected? -->
    <collection-method>Buccal swab</collection-method>

    <!-- Below follows the actual typing information for this specific sample -->
    <!-- For which gene family was this sample typed and on what date? -->
    <typing gene-family="HLA"
            date="2026-03-16">


      <!-- MINIMAL RECOMMENDATION: Allele assignment - IPD-IMGT/HLA Database version -->
      <!-- When were the alleles assigned? Which database was used? -->
```

```xml
    <allele-assignment date="2026-03-15"
                       allele-db="IPD-IMGT/HLA"
                       allele-version="3.48.0">

      <!-- MINIMAL RECOMMENDATION: Allele assignment - Genotype in GL String format (full resolution) -->
      <!-- What was the typing result? -->
      <glstring>HLA-B*15:03:01:02/HLA-B*15:03:01:08+HLA-B*50:02:01:01</glstring>
    </allele-assignment>


    <!-- MINIMAL RECOMMENDATION: Must contain elements: Typing Method -->
    <!-- What typing methodology was used? -->
    <typing-method>
      <sbt-ngs locus="HLA-B"
               test-id="HLA-NGS-B-ASSAY-2047"
               test-id-source="manufacturer">

        <!-- MINIMAL RECOMMENDATION: Must contain elements - kit/lot number -->
        <!-- Which reagents were used? -->
        <property name="kit-name"
                  value="NGS Kit Name"/>
        <property name="lot-number"
                  value="LOT-26A123"/>

        <!-- MINIMAL RECOMMENDATION: Must contain elements - Platform -->
        <!-- Which platform was used? -->
        <property name="platform"
                  value="Illumina NovaSeq"/>

        <raw-reads uri="123456789012_ABCDEFGHIJ_S01_L001_R1_001.fastq.gz"
                   format="fastq"
                   paired="true"
                   pooled="true"
                   availability="private"
                   adapter-trimmed="false"
                   quality-trimmed="false">
        </raw-reads>

        <raw-reads uri="123456789012_ABCDEFGHIJ_S01_L001_R2_001.fastq.gz"
                   format="fastq"
                   paired="true"
                   pooled="true"
                   availability="private"
                   adapter-trimmed="false"
                   quality-trimmed="false">
        </raw-reads>
      </sbt-ngs>
    </typing-method>
```

```xml

  <consensus-sequence date="2026-03-15">
    <reference-database name="ipd-imgt/hla"
                        description="IPD-IMGT/HLA Database"
                        version="3.48.0"
                        availability="public"
                        curated="true"
                        uri="http://www.ebi.ac.uk/ipd/imgt/hla">

      <!-- OPTIONAL RECOMMENDATION: HLA consensus sequences-->
      <!-- Which sequences were used as reference? What part of it? What is the accession number of the reference sequence in the IPD-IMGT/HLA database? -->
      <reference-sequence id="ref15"
                          name="B*15:03:01:02"
                          start="0"
                          end="3864"
                          accession="HLA14102"
                          uri="http://www.ebi.ac.uk/ipd/imgt/hla">
      </reference-sequence>

      <reference-sequence id="ref16"
                          name="B*50:02:01:01"
                          start="0"
                          end="3868"
                          accession="HLA00342"
                          uri="http://www.ebi.ac.uk/ipd/imgt/hla">
      </reference-sequence>
    </reference-database>

    <!-- MINIMAL RECOMMENDATION: Must contain elements: Exons targeted; Phasing -->
    <consensus-sequence-block reference-sequence-id="ref15"
                              start="0"
                              end="20"
                              strand="-1"
                              phase-set="0"
                              continuity="false"
                              expected-copy-number="1">
      <sequence>GATCAGGACGAAGTCCCAGG</sequence>
    </consensus-sequence-block>

    <consensus-sequence-block reference-sequence-id="ref15"
                              start="20"
                              end="1751"
                              strand="-1"
                              phase-set="1"
                              continuity="true"
                              expected-copy-number="1">
      <sequence>
      TCCCGGACGGGGCTCTCAGGGTCTCAGGCTCCGAGAGCCTTGTCTGCATTGGGGAGGCGCAGCGTTGGGGATTCCCCACTCCCACGAGTTTCACTTCTTCTCCCAACCTATGTCGGG
```

TCCTTCTTCCAGGATACTCGTGACGCGTCCCCATTTCCCACTCCCATTGGGTGTCGGGTGTCTAGAGAAGCCAATCAGTGTCGCCGGGGTCCCAGTTCTAAAGTCCCCACGCACCCA
CCCGGACTCAAAATCTCCTCAGACGCCGAGATGCGGGTCACGGCGCCCCGAACCGTCCTCCTGCTGCTCTCGGGAGCCCTGGCCCTGACCGAGACCTGGGCCGGTGAGTGCGGGGTC
GGCAGGGAAATGGCCTCTGTGGGGAGGAGCGAGGGGACCGCAGGCGGGGGCGCAGGACCCGGGGAGCCGCGCCGGGAGGAGGGTCGGGCGGGTCTCAGCCCCTCCTCGCCCCCAGGC
TCCCACTCCATGAGGTATTTCTACACCGCCATGTCCCGGCCCGGCCGCGGGGAGCCCCGCTTCATCTCAGTGGGCTACGTGGACGACACGCAGTTCGTGAGGTTCGACAGCGACGCC
GCGAGTCCGAGAGAGGAGCCGCGGGCGCCGTGGATAGAGCAGGAGGGGCCGGAGTATTGGGACCGGGAGACACAGATCTCCAAGACCAACACACAGACTTACCGAGAGAGCCTGCGG
AACCTGCGCGGCTACTACAACCAGAGCGAGGCCGGTGAGTGACCCCGGCCTGGGGCGCAGGTCACGACTCCCCATCCCCCACGTACGGCCCGGGTCGCCCCGAGTCTCCGGGTCCGA
GATCCGCCCCCCTGAGGCCGCGGGACCCGCCCAAACCCTCGACCGGCGAGAGCCCCAGGCGCGTTTACCCGGTTTCATTTTCAGTTGAGGCCAAAATCCCCGCGGGTTGGTCGGGGC
GGGGCGGGGCTCGGGGGACGGGGCTGACCGCGGGGCCTGGGCCAGGGTCTCACACCCTCCAGAGGATGTACGGCTGCGACGTGGGGCCGGACGGGCGCCTCCTCCGCGGGCATGACC
AGTCCGCCTACGACGGCAAGGATTACATCGCCCTGAACGAGGACCTGAGCTCCTGGACCGCGGCGGACACGGCGGCTCAGATCACCCAGCGCAAGTGGGAGGCGGCCCGTGAGGCGG
AGCAGCTGAGAGCCTACCTGGAGGGCCTGTGCGTGGAGTGGCTCCGCAGATACCTGGAGAACGGGAAGGAGACGCTGCAGCGCGCGGGTACCAGGGGCAGTGGGGAGCCTTCCCCAT
CTCCTATAGGTCGCCGGGGATGGCCTCCCACGAGAAGAGGAGGAAAATGGGATCAGCGCTAGAATGTCGCCCTCCCTTGAATGGAGAATGGCATGAGTTTTCCTGAGTTTCCTCTGA
GGGCCCCCTCTTCTCTCTAGGACAATTAAGGGATGACGTCTCTGAGGAAATGGAGGGGAAGACAGTCCCTAGGATAGTGATCAGGGGTCCCCTTTGACCCCTGCAGCAGCCTTGGGA
ACCGTGACTTTTCCTCTCAGGCCTTGTTCTCTGCCTCACACTCAGTGTGTTTGGGGCTCTGATTCCAGCACTTCTGAGTCACTTTACCTCCACTCAGATCAGGAGCAGAAGTCCCTG
TTCCCCGCTCAGAGACTCGAACTTTCCAATGAATAGGAGATTATCCCAGGTGCCTGCGTCCAGGCTGGTGTCTGGGTTCTGTGCCCCTTCCCT</sequence>
</consensus-sequence-block>

<consensus-sequence-block reference-sequence-id="ref15"
start="1751"
end="2529"
strand="-1"
phase-set="2"
continuity="true"
expected-copy-number="1">
<sequence>
ACCCCAGGTGTCCTGTCCATTCTCAGGCTGGTCACATGGGTGGTCCTAGGGTGTCCCATGAGAGATGCAAAGCGCCTGAATTTTCTGACTCTTCCCATCAGACCCCCCAAAGACACA
TGTGACCCACCACCCCATCTCTGACCATGAGGCCACCCTGAGGTGCTGGGCCCTGGGCTTCTACCCTGCGGAGATCACACTGACCTGGCAGCGGGATGGCGAGGACCAAACTCAGGA
CACCGAGCTTGTGGAGACCAGACCAGCAGGAGATAGAACCTTCCAGAAGTGGGCAGCTGTGGTGGTGCCTTCTGGAGAAGAGCAGAGATACACATGCCATGTACAGCATGAGGGGCT
GCCGAAGCCCCTCACCCTGAGATGGGGTAAGGAGGGGGATGAGGGGTCATATCTGTTCTCAGGGAAAGCAGGAGCCCTTCTGGAGCCCTTCAGCAGGGTCAGGGCCCCTCATCTTCC
CCTCCTTTCCCAGAGCCATCTTCCCAGTCCACCATCCCCATCGTGGGCATTGTTGCTGGCCTGGCTGTCCTAGCAGTTGTGGTCATCGGAGCTGTGGTCGCTACTGTGATGTGTAGG
AGGAAGAGCTCAGGTAGGGAAGGGGTGAGGGGTGGGGTCTGGGTTTTCTTGTCCCACTGGGGGTTTCAAGCCCCAGGTAGAAGTGTTCCCTGCCTCATTACTGGGAAGCAGCATCCA
CACAGGGGCTAACGCAGCCTGGGACCCTGTGTGCCAGCACTTACTCTTTTGTGCAGCACATGTGACAATGAAGGAC</sequence>
</consensus-sequence-block>

<consensus-sequence-block reference-sequence-id="ref15"
start="2529"
end="2991"
strand="-1"
phase-set="3"
continuity="true"
expected-copy-number="1">
<sequence>
AGATGTATCGCCTTGATGGTTGTGGTGTTGGGGTCCTGATTCCAGCATTCATGAGTCAGGGGAAGGTCCCTGCTAAGGACAGACCTTAGGAGGGCAGTTGGTCCAGGACCCACACTT
GCTTTCCTCGTGTTTCCTGATCCTGCCCTGGGTCTGTAGTCATACTTCTGGAAATTCCTTTTGGTTCCAAGACGAGGAGGTTCCTCTAAGATCTCATGGTCCTGCTTCCTCCCAGTC
CCCTCACAGGACATTTTCTTCCCACAGGTGGAAAAGGAGGGAGCTACTCTCAGGCTGCGTGTAAGTGGTGGGGGTGGGAGTGTGGAGGAGCTCACCCACCCCATAATTCCTCCTGTC
CCACGTCTCCTGCGGGCTCTGACCAGGTCCTGTTTTTGTTCTACTCCAGCCAGCGACAGTGCCCAGGGCTCTGATGTGTCTCTCACAGCTTGAAAAGGTGAGATTCTTGGG
</sequence>
</consensus-sequence-block>

<consensus-sequence-block reference-sequence-id="ref15"

```
                                start="3033"
                                end="3089"
                                strand="-1"
                                phase-set="3"
                                continuity="false"
                                expected-copy-number="1">
      <sequence>AGTGGGGAAAGGCCTGGGTAATGGAGATTCTTTGATTGGGATGTTTCGCGTGTGTC</sequence>
    </consensus-sequence-block>

    <consensus-sequence-block reference-sequence-id="ref15"
                                start="3089"
                                end="3326"
                                strand="-1"
                                phase-set="4"
                                continuity="true"
                                expected-copy-number="1">
      <sequence>
      GTGGGCTGTTCAGAGTGTCATCACTTACCATGACTAACCAGAATTTGTTCATGACTGTTGTTTTCTGTAGCCTGAGACAGCTGTCTTGTGAGGGACTGAGATGCAGGATTTCTTCAC
      GCCTCCCCTTTGTGACTTCAAGAGCCTCTGGCATCTCTTTCTGCAAAGGCACCTGAATGTGTCTGCGTCCCTGTTAGCATAATGTGAGGAGGTGGAGAGACAGCCCACCCTTGTGTC
      CAC</sequence>
    </consensus-sequence-block>

    <consensus-sequence-block reference-sequence-id="ref16" start="0" end="20" strand="-1" phase-set="0" continuity="false"
     expected-copy-number="1">
      <sequence>GATCAGGACGAAGTCCCAGG</sequence>
    </consensus-sequence-block>

    <consensus-sequence-block reference-sequence-id="ref16"
                                start="20"
                                end="1755"
                                strand="-1"
                                phase-set="1"
                                continuity="true"
                                expected-copy-number="1">
      <sequence>
      CCCCGGGCGGGGCTCTCAGGGTCTCAGGCTCCGAGGGCCGCGTCTGCAATGGGGAGGCGCAGCGTTGGGGATTCCCCACTCCCACGAGTTTCACTTCTTCTCCCAACCTATGTCGGG
      TCCTTCTTCCAGGATACTCGTGACGCGTCCCCATTTCCCACTCCCATTGGGTGTCGGGTGTCTAGAGAAGCCAATCAGCGTCGCCGTGGTCCCAGTTCTAAAGTCCCCACGCACCCA
      CCCGGACTCAGAATCTCCTCAGACGCCGAGATGCGGGTCACGGCACCCCGAACCGTCCTCCTGCTGCTCTCGGCGGCCCTGGCCCTGACCGAGACCTGGGCCGGTGAGTGCGGGTCG
      GCAGGGAAATGGCCTCTGTGGGGAGGAGCGAGGGGACCGCAGGCGGGGGCGCAGGACCCGGGGAGCCGCGCCGGGAGGAGGGTCGGGCGGGTCTCAGCCCCTCCTCGCCCCCAGGCT
      CCCACTCCATGAGGTATTTCCACACCGCCATGTCCCGGCCCGGCCGCGGGGAGCCCCGCTTCATCACCGTGGGCTACGTGGACGACACGCTGTTCGTGAGGTTCGACAGCGACGCCA
      CGAGTCCGAGGAAGGAGCCGCGGGCGCCATGGATAGAGCAGGAGGGGCCGGAGTATTGGGACCGGGAGACACAGATCTCCAAGACCAACACACAGACTTACCGAGAGAGCCTGCGGA
      ACCTGCGCGGCTACTACAACCAGAGCGAGGCCGGTGAGTGACCCCGGCCCGGGGCGCAGGTCACGACTCCCCATCCCCCACGTACGGCCCGGGTCGCCCCGAGTCTCCGGGTCCGAG
      ATCCGCCCCCCTGAGGCCGCGGGACCCGCCCAGACCCTCGACCGGCGAGAGCCCCAGGCGCGTTTACCCGGTTTCATTTTCAGTTGAGGCCAAAATCCCCGCGGGTTGGTCGGGGCG
      GGGCGGGGCGGGGCTCGGGGGACGGGGCTGACCGCGGGGCCTGGGCCAGGGTCTCACACTTGGCAGAGGATGTATGGCTGCGACCTGGGGCCCGACGGGCGCCTCCTCCGCGGGTAT
      AACCAGTTAGCCTACGACGGCAAGGATTACATCGCCCTGAACGAGGACCTGAGCTCCTGGACCGCGGCGGACACCGCGGCTCAGATCACCCAGCGCAAGTGGGAGGCGGCCCGTGAG
      GCGGAGCAGCTGAGAGCCTACCTGGAGGGCCTGTGCGTGGAGTCGCTCCGCAGATACCTGGAGAACGGGAAGGAGACGCTGCAGCGCGCGGGTACCAGGGGCAGTGGGGAGCCTTCC
      CCATCTCCTATAGGTCGCCGGGGATGGCCTCCCACGAGAAGAGGAGGAAAATGGGATCAGCGCTAGAATGTCGCCCTCCCTTGAATGGAGAATGGCATGAGTTTTCCTGAGTTTCCT
      CTGAGGGCCCCCTCTTCTCTCTAGGACAATTAAGGGATGACGTCTCTGAGGAAATGGAGGGGAAGTCAGTCCCTAGAATACTGATCAGGGGTCCCCTTTGACCCCTGCAGCAGCCTT
      GGGAACCGTGACTTTTCCTCTCAGGCCTTGTTCTCTGCCTCACACTCAGTGTGTTTGGGGCTCTGATTCCAGCACTTCTGAGTCACTTTACCTCCACTCAGATCAGGAGCAGAAGTC
```

```
    CCTGTTCCCCGCTCAGAGACTCGAACTTTCCAATGAATAGGAGATTATCCCAGGTGCCTGCGTCCAGGCTGGTGTCTGGGTTCTGTGCCCCTTCCCC</sequence>
  </consensus-sequence-block>

  <consensus-sequence-block reference-sequence-id="ref16"
                            start="1755"
                            end="2533"
                            strand="-1"
                            phase-set="2"
                            continuity="true"
                            expected-copy-number="1">
    <sequence>
    ACCCCAGGTGTCCTGTCCATTCTCAGGCTGGTCACATGGGTGGTCCTAGGGTGTCCCATGAGAGATGCAAAGCGCCTGAATTTTCTGACTCTTCCCATCAGACCCCCCAAAGACACA
    TGTGACCCACCACCCCATCTCTGACCATGAGGCCACCCTGAGGTGCTGGGCCCTGGGCTTCTACCCTGCGGAGATCACACTGACCTGGCAGCGGGATGGCGAGGACCAAACTCAGGA
    CACCGAGCTTGTGGAGACCAGACCAGCAGGAGATAGAACCTTCCAGAAGTGGGCAGCTGTGGTGGTGCCTTCTGGAGAAGAGCAGAGATACACATGCCATGTACAGCATGAGGGGCT
    GCCGAAGCCCCTCACCCTGAGATGGGGTAAGGAGGGGGATGAGGGGTCATATCTGTTCTCAGGGAAAGCAGGAGCCCTTCTGGAGCCCTTCAGCAGGGTCAGGGCCCCTCATCTTCC
    CCTCCTTTCCCAGAGCCATCTTCCCAGTCCACCATCCCCATCGTGGGCATTGTTGCTGGCCTGGCTGTCCTAGCAGTTGTGGTCATCGGAGCTGTGGTCGCTACTGTGATGTGTAGG
    AGGAAGAGCTCAGGTAGGGAAGGGGTGAGGGGTGGGGTCTGGGTTTTCTTGTCCCACTGGGGGTTTCAAGCCCCAGGTAGAAGTGTTCCCTGCCTCATTACTGGGAAGCAGCATCCA
    CACAGGGGCTAACGCAGCCTGGGACCCTGTGTGCCAGCACTTACTCTTTTGTGCAGCACATGTGACAATGAAGGAC</sequence>
  </consensus-sequence-block>

  <consensus-sequence-block reference-sequence-id="ref16"
                            start="2533"
                            end="2995"
                            strand="-1"
                            phase-set="3"
                            continuity="true"
                            expected-copy-number="1">
    <sequence>
    GGATGTATCGCCTTGATGGTTGTGGTGTTGGGGTCCTGATTCCAGCATTCATGAGTCAGGGGAAGGTCCCTGCTAAGGACAGACCTTAGGAGGGCAGTTGGTCCAGGACCCACACTT
    GCTTTCCTCGTGTTTCCTGATCCTGCCTTGGGTCTGTAGTCATACTTCTGGAAATTCCTTTTGGTTCCAAGACGAGGAGGTTCCTCTAAGATCTCATGGCCCTGCTTCCTCCCAGTC
    CCCTCACAGGACATTTTCTTCCCACAGGTGGAAAAGGAGGGAGCTACTCTCAGGCTGCGTGTAAGTGGTGGGGGTGGGAGTGTGGAGGAGCTCACCCACCCCATAATTCCTCCTGTC
    CCACGTCTCCTGAGGGCTCTGACCAGGTCCTGTTTTTGTTCTACTCCAGCCAGCGACAGTGCCCAGGGCTCTGATGTGTCTCTCACAGCTTGAAAAGGTGAGATTCTTGGG
    </sequence>
  </consensus-sequence-block>

  <consensus-sequence-block reference-sequence-id="ref16"
                            start="3037"
                            end="3093"
                            strand="-1"
                            phase-set="3"
                            continuity="false"
                            expected-copy-number="1">
    <sequence>AGTGGGGAAAGGCCTGGGTAATGGAGATTCTTTGATTGGGATGTTTCGCGTGTGTG</sequence>
  </consensus-sequence-block>

  <consensus-sequence-block reference-sequence-id="ref16"
                            start="3093"
                            end="3330"
                            strand="-1"
```

```xml
                                    phase-set="4"
                                    continuity="true"
                                    expected-copy-number="1">
          <sequence>
          GTGGGCTGTTCAGAGTGTCATCACTTACCATGACTAACCAGAATTTGTTCATGACTGTTGTTTTCTGTAGCCTGAGACAGCTGTCTTGTGAGGGACTGAGATGCAGGATTTCTTCAC
          GCCTCCCCTTTGTGACTTCAAGAGCCTCTGGCATCTCTTTCTGCAAAGGCACCTGAATGTGTCTGCGTCCCTGTTAGCATAATGTGAGGAGGTGGAGAGACAGCCCACCCTTGTGTC
          CAC</sequence>
        </consensus-sequence-block>

      </consensus-sequence>

    </typing>

  </sample>

</hml>
```
